# Betulinic Acid Exerts Cytotoxic Activity Against Multidrug-Resistant Tumor Cells via Targeting Autocrine Motility Factor Receptor (AMFR)

**DOI:** 10.3389/fphar.2018.00481

**Published:** 2018-05-15

**Authors:** Mohamed E. M. Saeed, Nuha Mahmoud, Yoshikazu Sugimoto, Thomas Efferth, Heba Abdel-Aziz

**Affiliations:** ^1^Department of Pharmaceutical Biology, Johannes Gutenberg University, Mainz, Germany; ^2^Division of Chemotherapy, Faculty of Pharmacy, Keio University, Tokyo, Japan; ^3^Medical and Clinical Affairs Phytomedicines, Steigerwald Arzneimittelwerk GmbH, Bayer Consumer Health, Darmstadt, Germany

**Keywords:** bioinformatics, cancer, drug resistance, microarray, pharmacogenomics, phytotherapy, triterpene, autocrine motility factor receptor (AMFR)

## Abstract

Betulinic acid (BetA) is a naturally occurring pentacyclic triterpene isolated from the outer bark of white-barked birch trees and many other medicinal plants. Here, we studied betulinic acid's cytotoxic activity against drug-resistant tumor cell lines. P-glycoprotein (*MDR1/ABCB1*) and BCRP (*ABCG2*) are known ATP-binding cassette (ABC) drug transporters that mediating MDR. ABCB5 is a close relative to ABCB1, which also mediates MDR. Constitutive activation of the EGF receptor is tightly linked to the development of chemotherapeutic resistance. BetA inhibited P-gp, BCRP, ABCB5 and mutation activated EGFR overexpressing cells with similar efficacy as their drug-sensitive parental counterparts. Furthermore, the mRNA expressions of ABCB1, BCRP, ABCB5 and EGFR were not related to the 50% inhibition concentrations (IC_50_) for BetA in a panel of 60 cell lines of the National Cancer Institute (NCI), USA. In addition to well-established MDR mechanisms, we attempted to identify other molecular mechanisms that play a role in mediating BetA's cytotoxic activity. For this reason, we performed COMPARE and hierarchical cluster analyses of the transcriptome-wide microarray-based mRNA expression of the NCI cell lines panel. Various genes significantly correlating to BetA's activity were involved in different biological processes, e.g., cell cycle regulation, microtubule formation, signal transduction, transcriptional regulation, chromatin remodeling, cell adhesion, tumor suppression, ubiquitination and proteasome degradation. Immunoblotting and *in silico* analyses revealed that the inhibition of AMFR activity might be one of the mechanisms for BetA to overcome MDR phenotypes. In conclusion, BetA may have therapeutic potential for the treatment of refractory tumors.

## Introduction

Betulinic acid (BetA) is a lupane-type triterpenoid firstly identified and isolated in the 18th century by Johann Tobias Lowitz from the outer bark of white-barked birch trees (Cichewicz and Kouzi, 2004). It can also be found in *Vitex negundo* (Chandramu et al., 2003), *Quisqualis fructus* (Woldemichael et al., 2003), *Berlinia grandiflora* (Enwerem et al., 2001), *Tetracentron sinense* (Yi et al., 2000), *Orthosiphon stamineus* (Tezuka et al., 2000), *Eucalyptus camaldulensis* (Siddiqui et al., 2000), *Syncarpa glomulifera* (Setzer et al., 2000), and *Ziziphus spec*. (Schühly et al., 1999). BetA possesses several biological activities including anti-inflammatory, anticancer, and anti-HIV activities (Cichewicz and Kouzi, 2004). Several studies have been carried out proving the role of BetA and its derivatives in HIV proteases and reverse transcriptase inhibition, suggesting that BetA is a promising candidate for further development to treat HIV (Mayaux et al., 1994; Pengsuparp et al., 1994; Li et al., 2003).

BetA inhibited proliferation and induced apoptosis in various cancer cell lines such as breast, prostate, brain, colon, and leukemia (Fulda et al., 1999; Raghuvar Gopal et al., 2005; Chintharlapalli et al., 2007; Jung et al., 2007; Tiwari et al., 2014). Moreover, BetA has shown *in vivo* anticancer activity in melanoma and prostate xenograft mouse models (Eiznhamer and Xu, 2004; Chintharlapalli et al., 2007). Interestingly, *in vivo* studies indicated that BetA has a high safety margin, as systemic side effects were not noticed at the dose range tested (Eiznhamer and Xu, 2004). BetA induced apoptosis through affecting mitochondrial membrane potential, leading to increased permeability transition pore openings and production of reactive oxygen species. Subsequently, the release of mitochondrial apogenic factors occurs activating caspases and forming DNA fragments (Fulda et al., 1997, 1998). Furthermore, BetA inhibited aminopeptidase N, an enzyme involved in angiogenesis and metastatic activity during tumor growth (Melzig and Bormann, 1998).

Alternative therapeutic approaches for cancer treatment are urgently needed due to resistance developed toward the vast majority of clinically established anticancer drugs. Thus, implementation of rational alternative medicine using natural products could be one of the choices to overcome drug resistance and to re-sensitize refractory cancer cells to treatment.

The factors involved in multidrug resistance (MDR) are classified into either host factors, in which decreased absorption and distribution with increased metabolism and excretion take place for certain drugs, or cancer cell genetic alterations, in which alternative pathways to evade apoptosis and triggered cell death are activated. Therefore, the design of MDR reversing agents should take into account the factors mentioned above to improve the cancer therapeutic approaches.

Autocrine motility factor receptor (AMFR) is an ubiquitin E3-ligase cell surface glycoprotein, also known as GP78, that is known to play a role in metastasis, tumor progression and recurrence. AMFR regulates cell motility signaling *in vitro* and metastasis *in vivo* (Onishi et al., 2003). Furthermore, AMFR is a key player in endoplasmic reticulum-associated degradation (ERAD), targeting misfolded or functionally denatured proteins for proteasome degradation (Halwani et al., 2015). Its ligand, autocrine motility factor (AMF), a motility-stimulating cytokine that is secreted by tumor cells, has been shown to regulate proliferation, tumor migration and apoptosis resistance (Silletti et al., 1991; Shimizu et al., 1999). Recently, the role of AMF in drug resistance has been demonstrated. Kho et al. affirmed that in human breast carcinoma the interaction of AMF with HER2 triggers HER2 phosphorylation and metalloprotease-mediated ectodomain shedding, activating PI3K and MAPK signaling and hinders trastuzumab effect (Kho et al., 2013). Several studies reported the overexpression of AMFR in different types of human cancer, including esophageal carcinoma, breast carcinoma, pulmonary cancer and melanoma (Tímár et al., 2002; Kaynak et al., 2005; Kojic et al., 2007; Wang et al., 2010a).

In the present work, BetA's anticancer activity against drug-resistant tumor cell lines was studied, in which either MDR-conferring ABC-transporters (P-glycoprotein, BCRP, and ABCB5) or mutation-activated EGFR were overexpressed. Furthermore, we investigated the transcriptome-wide mRNA expression profiles of tumor 60 cell lines by COMPARE and hierarchical cluster analyses, to determine multiple other unknown molecular determinants that affect the response of tumor cells to BetA. Using gene-hunting approach, one of the sensitivity determinants genes, AMFR, was selected as a target candidate for BetA. We present novel evidence via molecular docking and immunoblotting analysis that inhibition of AMFR activity might be one of the mechanisms for BetA to overcome MDR phenotypes.

## Materials and methods

### Cell lines

Drug sensitive CCRF-CEM and multidrug-resistant P-glycoprotein (P-gp)-overexpressing CEM/ADR5000 leukemic cells were cultured in RPMI-1640 medium supplemented with 10% FBS and 1% penicillin/streptomycin (Invitrogen, Darmstadt, Germany). Doxorubicin (5,000 ng/mL) was added to maintain overexpression of P-gp (MDR1, ABCB1) in resistant cells (Kimmig et al., 1990). Breast cancer cells transfected with a control vector (MDA-MB-231-pcDNA3) or with cDNA for the breast cancer resistance protein BCRP/ABCG2 (MDA-MB-231-BCRP clone 23) were cultured and maintained as reported (Doyle et al., 1998). Expression of ABCG2 in resistant cells was maintained by geneticin (800 ng/mL) (Efferth et al., 2003). Human HEK293-ABCB5 embryonic kidney cells transfected with another ABC-transporter, ABCB5, were propagated in DMEM medium supplemented with 10% FBS and 1% penicillin/streptomycin (Invitrogen) (Kawanobe et al., 2012). Non-transfected HEK293 cells served as control. Wild-type human glioblastoma multiform (GBM) U87MG cells and cells transfected with control mock vector or an expression vector harboring EGFR cDNA with a deletion in exons 2-7 (U87MGΔEGFR), were kindly provided by Dr. W. K. Cavenee (Ludwig Institute for Cancer Research, San Diego, CA) (Huang et al., 1997).

### Cytotoxicity assays

The resazurin (Promega, Mannheim, Germany) reduction assay was performed to assess cytotoxicity of BetA in a concentration range of 10^−5^-100 μM as previously described (Kuete and Efferth, 2013). The IC_50_ values have been calculated from dose response curves and resistance ratios were determined by dividing the IC_50_ of resistant cells by the IC_50_ of the corresponding parental cells. Each assay has been done thrice with six replicates for each concentration.

### COMPARE and hierarchical cluster analyses of microarray data

We performed COMPARE analysis for a transcriptome-wide search for correlations between gene expressions and BetA response (log_10_IC_50_ values) deposited at the NCI website (http://dtp.nci.nih.gov) to identify candidate genes mediate sensitivity and resistance, respectively, to BetA. This gene-hunting approach is based on Pearson's rank correlation test. To obtain COMPARE rankings, a scale index of correlation coefficients (*R*-values) has been generated.

Using the CIMMINER program (https://discover.nci.nih.gov/cimminer/), we performed agglomerative hierarchical cluster analysis (WARD method) to cluster the mRNA expression of genes identified by COMPARE analysis and a heatmap was prepared accordingly.

Pearson's correlation test was used to calculate significance values and rank correlation coefficients as a relative measure for the linear dependence of two variables. The χ^2^ test was done using the Excel program to proof the frequency distributions for pairs of observed and expected variables for dependencies obtained from cluster analysis/heat mapping.

### Western blotting analysis

Both sensitive and resistant breast cancer cell lines (10^6^ cells/well) were treated with varying concentrations of BetA, harvested after 24 h, and washed with PBS. Using M-PER™ mammalian protein extraction buffer (Thermo Fisher Scientific), the entire proteins were extracted from the cell lysates. Then, sodium dodecyl sulfate polyacrylamide gel electrophoresis was performed to isolate the proteins, the proteins were then transferred to polyvinylidene fluoride membranes (Ruti®-PVDF) (Millipore, Billerica, MA, USA) for western blotting. Five percent of bovine serum albumin was used to block the membranes and then the membranes were incubated with specific primary antibodies against AMFR (1:1,000) (Thermoscientific, Darmstadt, Germany) and β-actin (1:2,000) (Cell Signaling Technology, Frankfurt, Germany). The blots were probed with horseradish peroxidase-linked IgG secondary antibodies (1:2,000) for 2 h at room temperature. Finally, LuminataTM Classico Western HRP substrate (Merck Millipore, Schwalbach, Germany) was added for 5 min in the dark. Alpha Innotech FluorChem Q system (Biozym, Oldendorf, Germany) was used for documentation and band analysis.

### Molecular docking

Molecular docking was done in an approach previously reported by us (Saeed et al., 2018). Briefly, the PDB files of different AMFR cytosolic C terminal domains and AMF were obtained from protein data bank (registered ids are shown in **Table 3**). Using AutodockTools-1.5.6rc3, the proteins' PDB files were converted to PDBQT format and set as the macromolecule, upon which docking is to be performed. 2D structures of the BetA was constructed and later converted to 3D structures using Corina Online Demo. A grid box was allocated to define docking spaces upon the macromolecule. Energies for each atom type in the ligand were calculated at each grid point using Autogrid 4.2. These calculated energies were later used to predict binding affinity of BetA. Docking was carried out using Autodock 4.2 with 250 runs and 2.5 million evaluations for each cycle via the Lamarckian algorithm. Lowest binding energies were retrieved from the correspondent dlg file and amino acids were analyzed by Autodock Tools. Images were created using Visual Molecular Dynamics VMD.

## Results

### Cytotoxic response of betulinic acid in drug-resistant tumor cell lines

**Figure 1:**
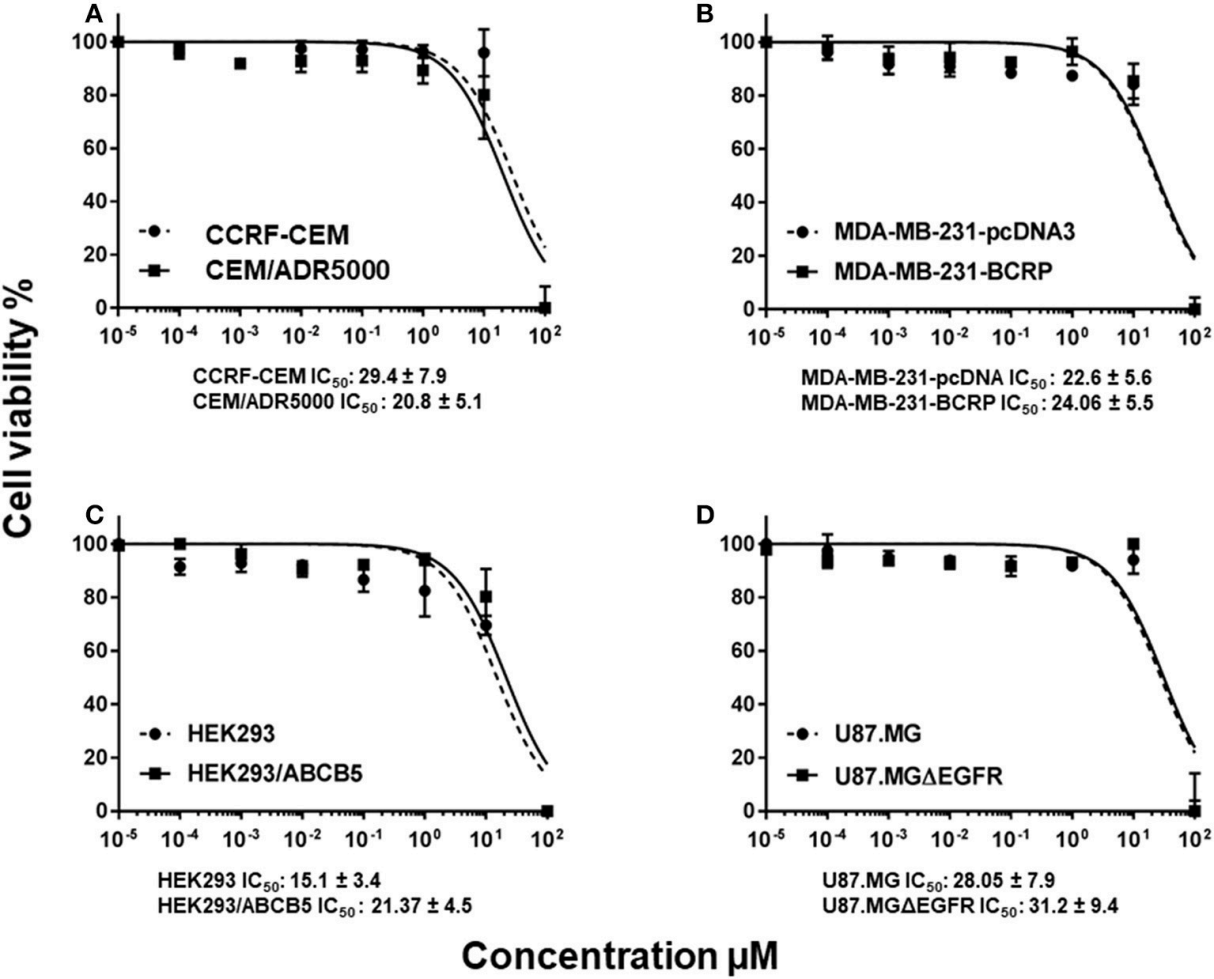
Dose response curves of BetA. **(A)** Cytotoxicity of BetA toward drug-sensitive parental CCRF-CEM tumor cells and their P-glycoprotein (MDR1/ABCB1)-expressing, multidrug-resistant subline, CEM/ADR5000. **(B)** Cytotoxicity of BetA toward MDA-MB-231-pc DNA cells and their BCRP-transduced subline, MDA-MB-231-BCRP as determined by resazurin assays. **(C)** Cytotoxicity of BetA toward HEK293 cells and their ABCB5-transfectant subline, HEK293/ABCB5 as determined by resazurin assays. **(D)** Cytotoxicity of BetA U87MG cells and their EGFR- transduced subline U87MGΔEGFR, as determined by resazurin assays.

In order to study whether or not the classical MDR mechanisms impede the cytotoxic activity of BetA toward cancer cells, we investigated multidrug-resistant P-glycoprotein (MDR1/ABCB1)-overexpressing CEM/ADR5000 cells and drug-sensitive parental CCRF-CEM cells using a resazurin assay. No cross-resistance of the CEM/ADR5000 cells was observed (0.8-fold, Figure 1A).

As other cell models that overexpress ABC transporters, we tested MDA-MB-231 cells transfected with BCRP/ABCG2 and HEK-293 cells transfected with ABCB5. Both cell lines were sensitive to BetA, if compared with their drug-sensitive parental cells (0.9 and 1.2-fold, respectively (Figures 1B,C).

U87.MG cells transfected with a deletion-activated EGFR cDNA showed similar sensitivity to BetA than their wild-type counterpart (Figure 1D).

### Analysis of classical drug resistance mechanisms for betulinic acid

Pearson's correlation coefficient was used to correlate the expression data of different genes that are responsible for anticancer drug resistance (ABCB1, ABCC1, ABCG2 and ABCB5, EGFR, and mutated TP53) with the log_10_IC_50_ values of the NCI cell line panel for BetA. We analyzed microarray, protein array, cDNA sequencing, RT-PCR-based mRNA expressions for the mentioned genes. As shown in Table 1, these parameters did not significantly correlate with the log_10_IC_50_ values for BetA, indicating that its cellular cytotoxicity was not affected by these drug resistance mechanisms. For the validity of our approach, positive control drugs have been used, which were strongly correlated with their corresponding mechanisms of resistance (daunorubicin for ABCB1, maytansine for ABCB5, vinblastine for ABCC1, pancratistatin for ABCG2, erlotinib for EGFR and 5-fluorouracil for mutated TP53; Table 1).

**Table 1:** Correlation of log_10_IC_50_ values for BetA to drug resistance mechanisms (ABCB1, ABCB5, ABCC1, ABCG2, EGFR, TP53) in the NCI cell line panel.

		**Betulinic acid**	**Control drug**
		**(log_10_ IC_50_, M)**	**(log_10_ IC_50_, M)**
**ABCB1 expression**			**Daunorubicin**
7q21 (chromosomal	*R*-value	−0.004	^*^0.597
Locus of ABCB1 gene)	*P*-value	0.489	^*^4.82 × 10^−6^
ABCB1 expression	*R*-value	0.036	^*^0.684
(Microarray)	*P*-value	0.394	^*^1.57 × 10^−8^
ABCB1 expression	*R*-value	0.153	^*^0.579
(RT-PCR)	*P*-value	0.142	^*^4.19 × 10^−6^
Rhodamine 123	*R*-value	0.076	^*^0.544
Accumulation	*P*-value	0.286	^*^1.51 × 10^−5^
**ABCB5 expression**			**Maytansine**
ABCB5 expression	*R*-value	0.052	^*^0.454
(Microarray)	*P*-value	0.347	^*^6.67 × 10^−4^
ABCB5 expression	*R*-value	0.164	^*^0.402
(RT-PCR)	*P*-value	0.105	^*^0.0034
**ABCC1 expression**			**Vinblastine**
DNA gene	*R*-value	0.148	^*^0.429
Copy number	*P*-value	0.131	^*^0.001
ABCC1 expression	*R*-value	−0.070	^*^0.399
(Microarray)	*P*-value	0.302	^*^0.002
ABCC1 expression	*R*-value	−0.091	0.299
(RT-PCR)	*P*-value	0.269	^*^0.036
**ABCG2 expression**			**Pancratistatin**
ABCG2 expression	*R*-value	−0.092	^*^0.329
(Microarray)	*P*-value	0.246	^*^0.006
ABCG2 expression	*R*-value	−0.051	^*^0.346
(Western blot)	*P*-value	0.352	^*^0.004
**EGFR expression**			**Erlotinib**
EGFR gene	*R*-value	−0.036	−0.245
Copy number	*P*-value	0.394	^*^0.029
EGFR expression	*R*-value	0.192	^*^−0.458
(Microarray)	*P*-value	0.071	^*^1.15 × 10^−4^
EGFR expression	*R*-value	0.203	^*^0.409
(RNAse protection)	*P*-value	0.064	^*^7.08 × 10^−4^
EGFR expression	*R*-value	−0.025	^*^−0.376
(Protein array)	*P*-value	0.425	^*^0.001
**TP53 mutation**			**5–Fluorouracil**
TP53 mutation	*R*-value	−0.066	^*^−0.502
(cDNA sequencing)	*P*-value	0.312	^*^3.50 × 10^−5^
TP53 function	*R*-value	0.012	^*^−0.436
(Yeast functional assay)	*P*-value	0.464	^*^5.49 × 10^−4^

* *P < 0.05 and R > 0.3 (or R < −0.3)*.

#### Drug class profiling

**Figure 2:**
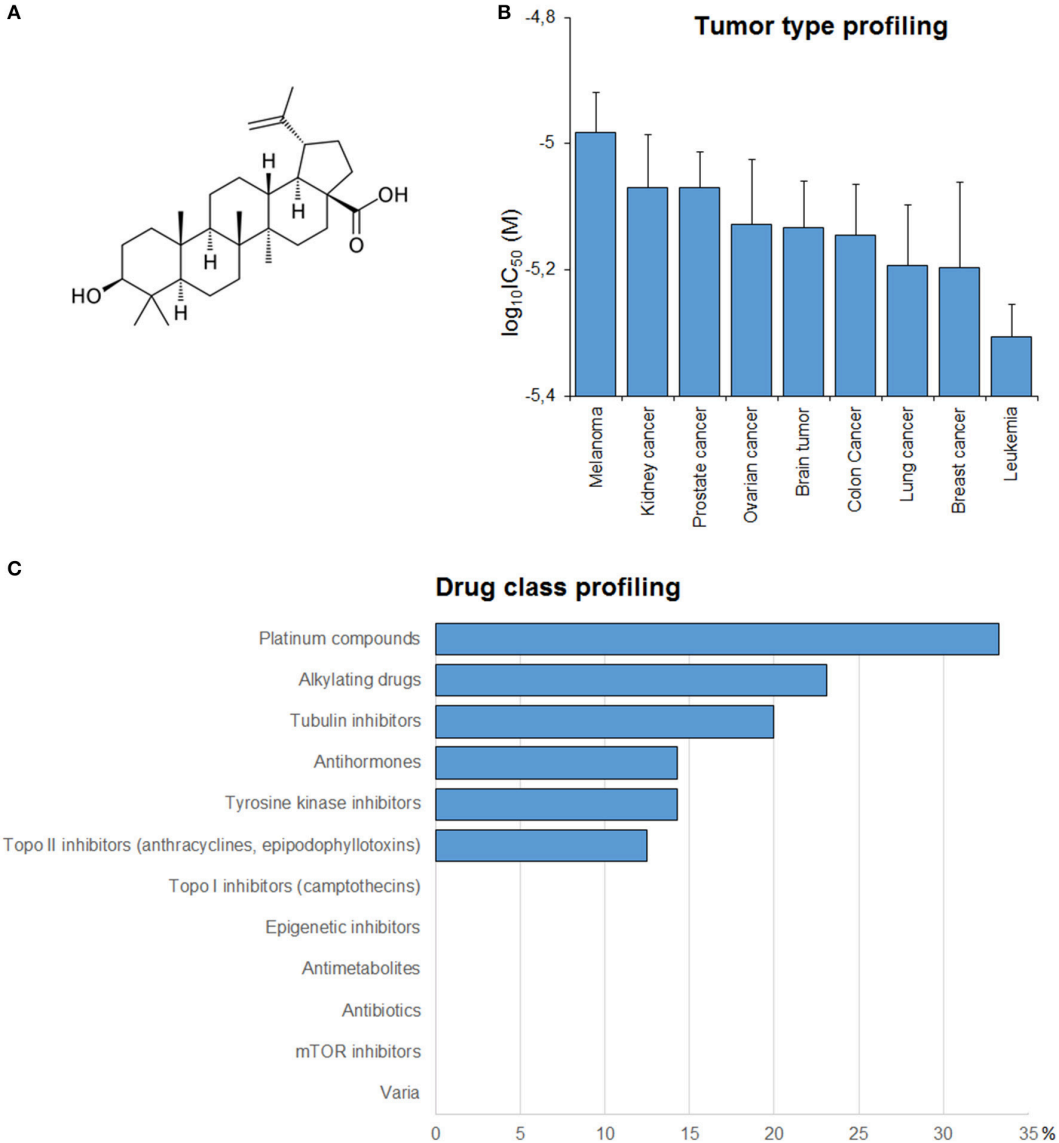
**(A)** Chemical structure of BetA. **(B)** Mean log_10_IC_50_ values for BetA of the NCI cell lines, and S.E.M. log_10_IC_50_ values were grouped according to the tumor type of the cell lines. **(C)** Percentage of classes of established anticancer drugs, whose log_10_IC_50_ values correlate with those for BetA.

In order to get a clue about possible modes of action of BetA (Figure 2A), BetA's log_10_IC_50_ values of the NCI cell lines were correlated with standard anticancer drugs (Figure 2C). The cellular response of platinum compounds, alkylating agents, tubulin inhibitors and tyrosine kinase inhibitors were significantly correlated with those of BetA.

#### Tumor-type dependent response toward betulinic acid

If the average log_10_IC_50_ values over the entire range of 60 cell lines were diversified regarding their tumor types, Melanoma cell lines were most resistant toward BetA, whereas leukemia cell lines were most sensitive (Figure 2B).

#### Microarray-based expression profiling to predict sensitivity and resistance to betulinic acid

To figure out genes that are mediated sensitivity or resistance of cancer cells toward BetA, we obtained the transcriptome-wide mRNA expressions of the NCI cells and correlated them with the log_10_IC_50_ values for BetA. Using Pearson's rank correlation, we carried out a transcriptome-wide COMPARE analysis to determine genes, whose mRNA expression directly or inversely correlated with the log_10_IC_50_ values for BetA. Fourty genes were identified, half of them were directly and the other half were inversely correlated to the log_10_IC_50_ values for BetA (Table 2). The proteins encoded by these genes have diverse biological functions (Table 2).

**Table 2:** Correlation of constitutive mRNA expression of genes identified by COMPARE analysis with log_10_IC_50_ values for BetA of the NCI tumor cell lines.

**COMPARE coefficient**	**Experiment ID**	**Gene symbol**	**Name**	**Function**
0.497	GC28294	*CHEK2*	CHK2 checkpoint homolog (S. pombe) RNA	Regulates cell cycle checkpoint arrest through phosphorylation of CDC25A, CDC25B, and CDC25C, inhibiting their activity.
0.428	GC33596	*CDC25C*	Cell division cycle 25 homolog C (S. pombe) RNA	This gene encodes a conserved protein that plays a key role in the regulation of cell division.
0.425	GC29591	*FBL*	Fibrillarin RNA	Also acts as a protein methyltransferase by mediating methylation of Gln-105 of histone H2A (H2AQ104me).
0.424	GC30713	*NVL*	Nuclear VCP-like RNA	May play a role in 60S ribosomal subunit biogenesis.
0.411	GC35294	*TPM2*	Tropomyosin 2 (beta) RNA	Plays a central role, in association with the troponin complex, in the calcium dependent regulation of vertebrate striated muscle contraction.
0.405	GC37151	*CCDC9*	Coiled-coil domain containing 9 RNA	Not available
0.403	GC39011	*LIN37*	Lin-37 homolog (C. elegans) RNA	Not available
0.391	GC27490	*ADAM3A*	ADAM metallopeptidase domain 3A RNA	Anchored cell surface adhesion protein.
0.389	GC37907	*KIFC1*	Kinesin family member C1 RNA	Minus end-directed microtubule-dependent motor required for bipolar spindle formation.
0.387	GC31813	*LSM2*	LSM2 homolog, U6 small nuclear RNA associated (S. cerevisiae) RNA	May be involved in pre-mRNA splicing.
0.386	GC29011	*FAM50A*	Family with sequence similarity 50, member A RNA	May be a DNA-binding protein or transcriptional factor.
0.384	GC28346	*DNTTIP2*	Deoxynucleotidyltransferase, terminal, interacting protein 2 RNA	May function as a chromatin remodeling protein.
0.384	GC31325	*LAGE3*	L antigen family, member 3 RNA	The complex is probably involved in the transfer of the threonylcarbamoyl moiety of threonylcarbamoyl-AMP (TC-AMP) to the N6 group of A37.
0.382	GC31480	*WDR67*	WD repeat domain 67 RNA	Not available
0.379	GC39257	*CBX5*	Chromobox homolog 5 RNA	Component of heterochromatin that recognizes and binds histone H3 tails methylated at Lys-9 (H3K9me), leading to epigenetic repression.
0.378	GC33227	*PTP4A2*	Protein tyrosine phosphatase type IVA, member 2 RNA	Protein tyrosine phosphatase which stimulates progression from G1 into S phase during mitosis.
0.377	GC39416	*LY6H*	Lymphocyte antigen 6 complex, locus H RNA	May play a role in the intracellular trafficking of alpha-7-containing nAChRs and may inhibit their expression at the cell surface.
0.375	GC27749	*CKS1B*	CDC28 protein kinase regulatory subunit 1B RNA	Binds to the catalytic subunit of the cyclin dependent kinases and is essential for their biological function.
0.375	GC38283	*GNG5*	Guanine nucleotide binding protein (G protein), gamma 5 RNA	Guanine nucleotide-binding proteins (G proteins) are involved as a modulator or transducer in various transmembrane signaling systems.
0.372	GC31629	*CCBL2*	Cysteine conjugate-beta lyase 2 RNA	Not available
−0.452	GC30666	*IQCK*	IQ motif containing K RNA	Not available
−0.417	GC27424	*LITAF*	Lipopolysaccharide-induced TNF factor RNA	EGFR and ERGG3 for lysosomal degradation, and thereby helps downregulate downstream signaling cascades.
−0.4	GC28487	*GNG12*	Guanine nucleotide binding protein (G protein), gamma 12 RNA	Guanine nucleotide-binding proteins (G proteins) are involved as a modulator or transducer in various transmembrane signaling systems.
−0.397	GC35009	*AP3S2*	Adaptor-related protein complex 3, sigma 2 subunit RNA	It facilitates the budding of vesicles from the Golgi membrane and may be directly involved in trafficking to lysosomes.
−0.389	GC30667	*IQCK*	IQ motif containing K RNA	Not available
−0.387	GC29994	*GRIK2*	Glutamate receptor, ionotropic, kainate 2 RNA	L-glutamate acts as an excitatory neurotransmitter at many synapses in the central nervous system.
−0.383	GC29004	*CEP170B*	Centrosomal Protein 170B	Plays a role in microtubule organization.
−0.381	GC29545	*PPP2R4*	Protein phosphatase 2A activator, regulatory subunit 4 RNA	Acts as a regulatory subunit for serine/threonine-protein phosphatase 2A.
−0.378	GC31014	*PTPRJ*	Protein tyrosine phosphatase, receptor type, J RNA	Plays a role in cell adhesion, migration, proliferation and differentiation.
−0.378	GC37170	*PKD2*	Polycystic kidney disease 2 (autosomal dominant) RNA	Functions as a cation channel involved in fluid-flow mechanosensation by the primary cilium in renal epithelium.
−0.374	GC38895	*ZNF652*	Zinc finger protein 652 RNA	Functions as a transcriptional repressor.
−0.372	GC28476	*AMFR*	Autocrine motility factor receptor RNA	E3 ubiquitin-protein ligase that mediates the polyubiquitination of a number of proteins such as CD3D, CYP3A4, CFTR and APOB for proteasomal degradation.
−0.368	GC34353	*ERN1*	Endoplasmic reticulum to nucleus signaling 1 RNA	This protein functions as a sensor of unfolded proteins in the endoplasmic reticulum (ER) and triggers an intracellular signaling pathway termed the unfolded protein response (UPR).
−0.364	GC32085	*SASH1*	SAM and SH3 domain containing 1 RNA	May have a role in a signaling pathway. Could act as a tumor suppressor.
−0.354	GC37023	*CLCN5*	Chloride channel 5 RNA	May play an important role in renal tubular function.
−0.354	GC29372	*NAGPA*	N-acetylglucosamine-1-phosphodiester alpha-N-acetylglucosaminidase RNA	Catalyzes the second step in the formation of the mannose 6-phosphate.
−0.354	GC33773	*ERBB3*	V-erb-b2 erythroblastic leukemia viral oncogene homolog 3 (avian) RNA	Tyrosine-protein kinase that plays an essential role as cell surface receptor for neuregulins.
−0.352	GC36873	*WWTR1*	WW domain containing transcription regulator 1 RNA	plays a pivotal role in organ size control and tumor suppression by restricting proliferation and promoting apoptosis.
−0.351	GC27626	*KANK1*	KN motif and ankyrin repeat domains 1 RNA	Involved in the control of cytoskeleton formation by regulating actin polymerization.
−0.351	GC35063	*SLC22A5*	Solute carrier family 22 (organic cation/carnitine transporter), member 5 RNA	The encoded protein is a plasma integral membrane protein which functions both as an organic cation transporter and as a sodium-dependent high affinity carnitine transporter. The encoded protein is involved in the active cellular uptake of carnitine.

**Figure 3:**
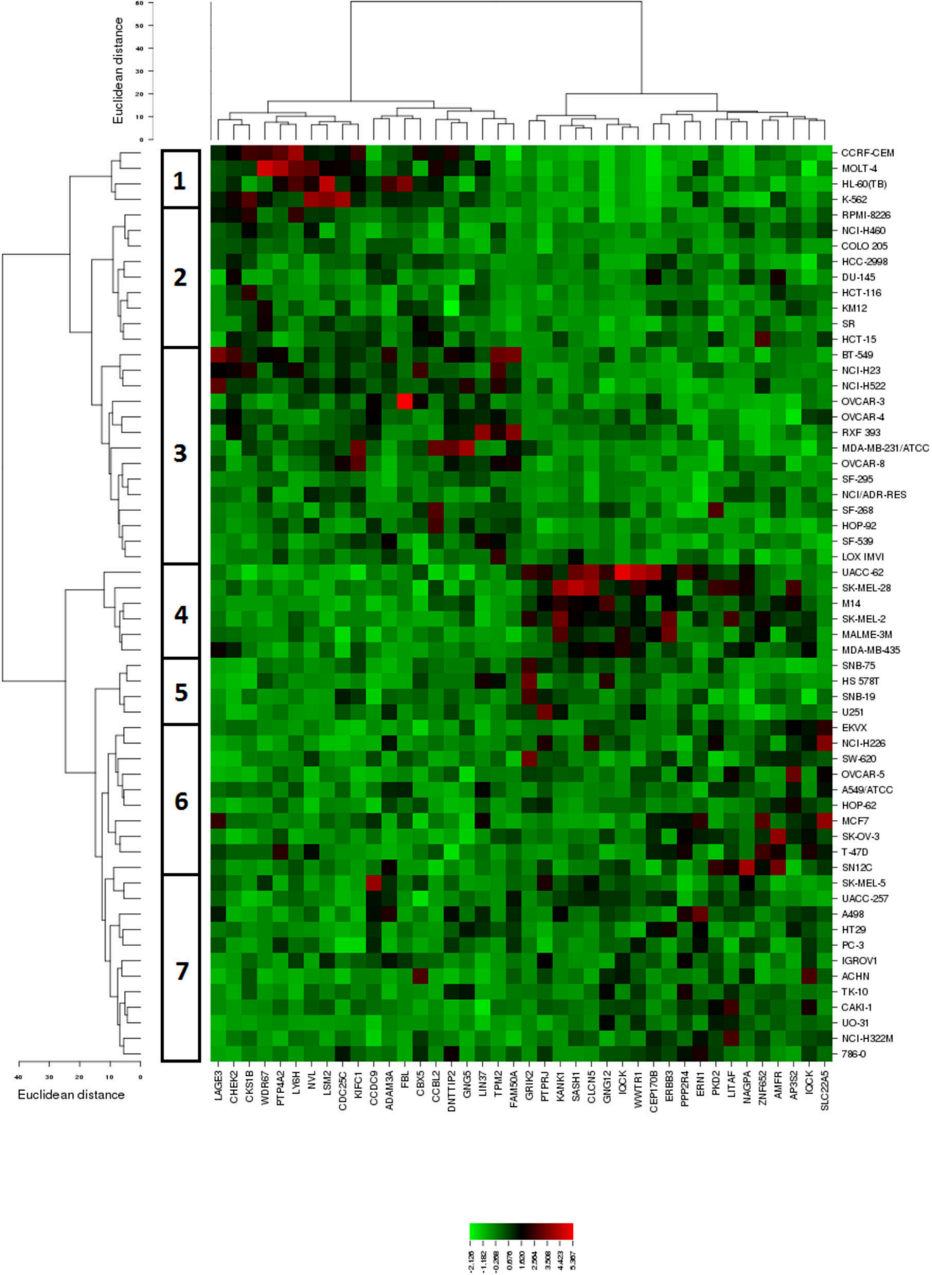
Dendrograms and heatmap of BetA obtained by hierarchical cluster analyses of NCI cells line panel and genes whose mRNA expression directly or inversely correlated with the log_10_IC_50_ values for BetA. The dendrogram on the left shows the clustering of cell lines.

The mRNA expression values of all NCI cell lines for the genes listed in Table 2 were subsequently subjected to hierarchical cluster analysis, in order to find out, whether clusters of cell lines could be identified with similar behavior after treatment with BetA. The dendrogram of the cluster analysis showed seven main branches in the cluster tree that depicted in the heatmap (Figure 3). As a next step, the log_10_IC_50_ values for BetA, which were not included in the cluster analysis, were assigned to the corresponding position of the cell lines in the cluster tree. The distribution among the seven clusters was significantly different from each other (*P* = 0.003). Clusters 5, 6, and 7 contained in its majority of cell lines sensitive to BetA, whereas clusters 1, 2, 3, and 4 contained in its majority resistant ones.

### Western blotting analysis

**Figure 4:**
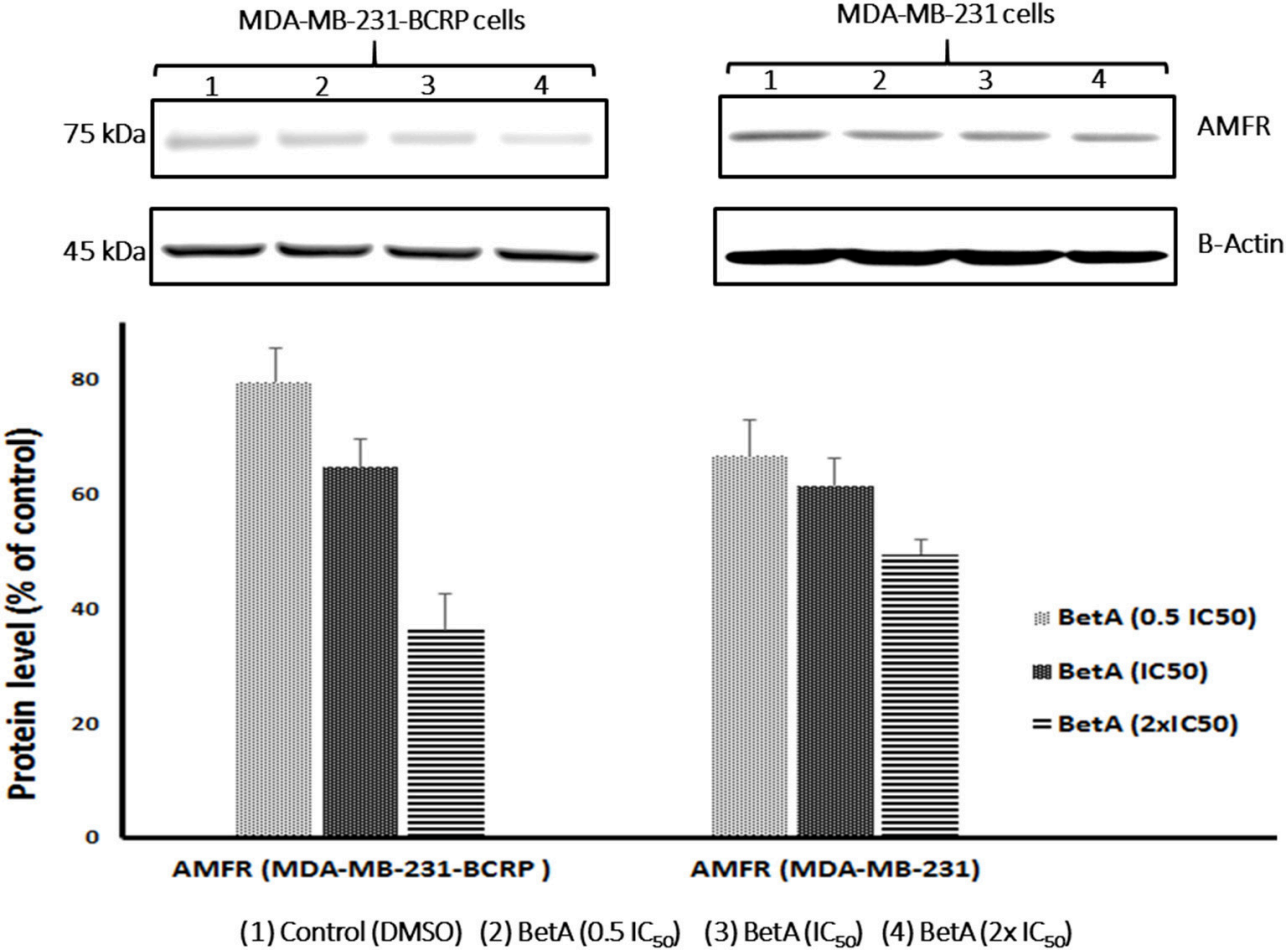
Western blot analysis of the effect of BetA on MDA-MB-231-BCRP and MDA-MB-231-pcDNA breast cancer cells. Evaluation of the AMFR expressions. β-actin was used as loading control. Bands were normalized to β-actin in order to obtain numerical values (Mean ± SEM).

The role of AMFR in drug resistance and tumor progression is well affirmed (Kho et al., 2013). Interestingly, better correlation between the expression values of AMFR and log_10_IC_50_ values for BetA in sensitive NCI cell lines was observed. This prompted us to investigate whether the expression of AMFR in our MDR cells model will be affected when treated with BetA or not. Therefore, we performed western blot analysis after treating breast cancer cell lines with varying concentration of BetA. The results showed that BetA was able to inhibit expression of AMFR in a dose dependent manner (Figure 4).

### Molecular docking

**Figure 5:**
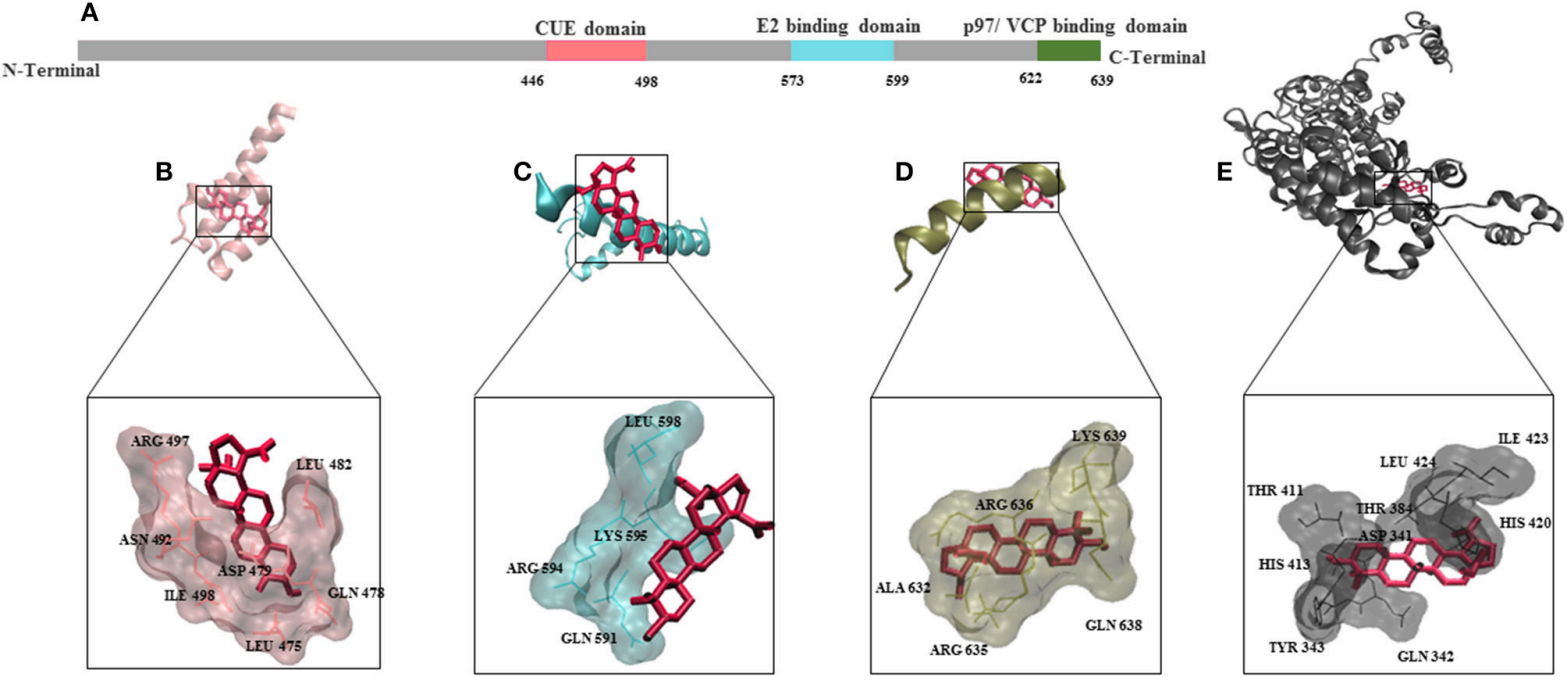
Molecular docking for BetA to AMFR (Autocrine Motility Factor Receptor) and its ligand AMF. **(A)** Schematic aligning structure of AMFR transmembrane domains at the N-terminal and cytosolic domains at C-terminal tail. **(B)** CUE domain functions to help substrate binding for ubiquitination. **(C)** E2 ubiquitin-conjugating enzyme binding domain. **(D)** p97/VCP binding domain that participates in the final step of endoplasmic reticulum-associated degradation. **(E)** Autocrine Motility factor ligand. The proteins were depicted in a new carton format, whereas BetA was represented in dynamic bond format with red color.

To understand the mode of binding of AMFR and its ligand AMF to BetA, we performed molecular docking analysis. BetA was docked on three different domains which are of crucial importance for AMFR function (CUE domain functions to help substrate binding for ubiquitination, E2 ubiquitin-conjugating enzyme binding domain, and p97/VCP binding domain that participates in the final step of endoplasmic reticulum-associated degradation). In addition to its ligand AMF. As shown in Table 3, the BetA bound to the three different domains in similar affinity (≈ −6.5 kcal/mol), whereas, it showed higher affinity to AMF with binding energy of −7.26 kcal/mol. The corresponding docking positions of BetA into binding pockets of AMFR domains and AMF are depicted in Figure 5.

**Table 3:** Molecular docking for BetA to different AMFR domains and its ligand AMF.

**Macromolecules**	**PDB ID**	**Lowest binding energy kcal/mol**	**pKi (μM)**	**AA involved in H-bonds**
**The CYTOSOLIC C-TERMINAL TAIL OF AMFR INCLUDES**				
Ubiquitin binding CUE motif	4G3O	−6.69 ± 0.07	12.45 ± 1.57	Arg 497, Gln478
E2 binding domain	4LAD	−6.36 ± 0.02	21.26 ± 0.36	Lys 595, Gln 591
p97/VCP binding domain	3TIW	−6.73± ≤ 0.01	11.66 ± 0.05	Arg 636
**LIGAND FOR AMFR**				
AMF/PGI	1NUH	−7.22 ± 0.06	5.14 ± 0.57	Thr 411

*Shown are lowest binding energy, predicted inhibition constant (Pki), amino acids (AA) involved in hydrogen bonding. Each docking experiment has been repeated three times*.

## Discussion

In this study, we investigated the cytotoxic activity of BetA against multidrug-resistant cancer cells and determined the molecular mechanisms associated with either sensitivity or resistance of cancer cells toward BetA. Therefore, we treated cancer cell lines expressing different MDR conferring genes (P-glycoprotein, BCRP, ABCB5 and mutation-activated EGFR) with BetA. To determine the molecular mechanisms, we mined the NCI's Developmental Therapeutics Program database for the documented screening of BetA with a panel of 60 cancer cell lines. The rationale of this approach was to define genes, whose expressions correlated to the pattern of cellular responsiveness to BetA. The genes assessed by this approach belonged to diverse classes, e.g. oncogenes, tumor suppressor genes, drug resistance mediating transporters, heat shock proteins, telomerase, cytokine receptors, molecules of the cell cycle and apoptotic pathways, DNA repair enzymes, components of cytoskeleton, intracellular signaling molecules, and enzymes of metabolism (Monga and Sausville, 2002).

Various mechanisms contribute to MDR in cancer cells. The dominant and possibly the most scrutinized one is drug efflux by a large superfamily of ATP-dependent efflux pumps, i.e., the ATP binding cassette (ABC) transporters (Ueda et al., 1986; Doyle et al., 1998). This superfamily belongs to one of the largest and most distributed superfamilies through all phyla, from prokaryotes to humans (Genovese et al., 2017). In normal eukaryotic cells, most ABC superfamily members export metabolites and xenobiotics outside cell membranes. Opportunistically, tumors take advantage of this normal function by overexpressing ABC transporters, subsequently, enhancing the expelling of chemotherapeutic drugs outside of the cells. Interestingly, the three tumor cell lines that overexpress three ABC transporters, P-gp, BCRP and ABCB5 did not confer resistance to BetA. These results showed that BetA is not a substrate of any of the three-abovementioned ABC transporters and indicate that refractory tumors overexpressing these transporters may effectively respond to BetA.

The epidermal growth factor receptor (EGFR) family activates signaling pathways regulating cellular proliferation, angiogenesis initiation, apoptosis inhibition and survival, which subsequently results in increasing tumor masses and chemotherapy refractoriness. In glioblastoma tumors, the common EGFR mutation is in frame deletion of exon 2–7 resulting in ligand-binding domain deletion of the EGFR (Nishikawa et al., 1994; Shinojima et al., 2003). This deletion causes constitutive activation of the receptor in the absence of ligand binding (Kuan et al., 2000; Arteaga, 2002). The auto-activated receptor phosphorylates tyrosine in the intracellular domain of the receptor, leading to activation of downstream signaling cascades. We tested the BetA effect on tumor cells transduced with mutated EGFR gene (resistant) and its sensitive parental cell line. BetA killed both cell lines at the same concentrations, showing that ΔEFGR does not confer resistance toward BetA.

Since the established MDR mechanisms investigated in this study were not involved in resistance toward BetA, we performed a microarray-based transcriptome wide screening of genes using COMPARE analysis, whose mRNA expression correlated with the log_10_IC_50_ values for BetA. Genes from diverse biological groups were identified to be correlated with the log_10_IC_50_ values for BetA, e.g., genes involved in cell cycle regulation and microtubule formation (*CHEK2, CDC25C, KIFC1, PTP4A2, CKS1B, CEP170B*), signal transduction and transcriptional regulation (*GNG5, GNG12, PPP2R4, ERBB3, ZNF652, FAM50A*), protein synthesis (*NVL, LSM2, ERN1, KANK1*), chromatin remodeling (*FBL, DNTTIP2, CBX5*), cell adhesion (*ADAM3A, PTPRJ*), tumor suppression (*SASH1, WWTR1*), ubiquitination and proteasome degradation (*AMFR, LITAF*). All of these mechanisms foster tumor progression.

The expression of AMFR is reported to correlate with solid tumors staging and survival rates (Wang et al., 2010a). As an ubiquitin E3 ligase protein, AMFR targets various proteins for degradation whose expression have an impact in cancer progression. For instance, the downregulation of AMFR-mediated ubiquitination of heat-shock proteins (HSPs) suppresses the metastasis of breast cancer (Chang et al., 2016). Worth to mention, the activation of AMFR by AMF can stimulate a signaling cascade, dependent on protein kinase C, and upregulates the Rho-like GTPase, RhoA and RhoC (Tsutsumi et al., 2002). Rho-associated, coiled-coil containing protein kinase 2 (ROCK2), a member of the RhoC family, functions as molecular determinants in several cellular functions, including proliferation, apoptosis and metastasis (Wang et al., 2010b). Apoptosis resistance is one of the prosurvival mechanisms for cancer cells and mediates the MDR to established chemotherapeutic agents. Moreover, it has been reported that autophagy promoted the development of paclitaxel and vinorelbine in breast cancer cells through inhibition of apoptosis (Sun et al., 2015). Wang et al. proved that overexpression of AMFR increased the levels of anti-apoptotic protein, Bcl-2, whereas downregulation of AMFR led to a significant decrease in the expression of Bcl-2 and increased early apoptosis (Wang et al., 2015). Furthermore, it has been shown that the cytokine, AMF, induced Apaf-1 and caspase-9 downregulation, leading to the apoptotic resistant phenotype in malignant cells (Haga et al., 2003). Considering the before mentioned studies, AMFR could be an attractive target for refractory and metastatic cancers therapy. Halwani et al., have coupled paclitaxel with AMF to target AMFR, they demonstrated that AMF represents a useful delivery vehicle for paclitaxel to AMFR overexpressing cancer cells *in vitro* and *in vivo*. The AMF–paclitaxel conjugate inhibited colon and breast cancer cells proliferation more effectively than free paclitaxel. Intra-tumoral injection of the AMF–paclitaxel conjugate also induced more effective tumor regression and increased survival in K1735M1 and B16-F1/paclitaxel resistant mouse melanoma models (Halwani et al., 2015). Intriguingly, BetA was able to inhibit AMFR in a dose dependent manner. Additionally, our molecular docking analysis revealed that BetA has higher affinity to bind AMF, suggesting that the AMF coupling might be an attractive approach to potentiate BetA's activity in tumor cells.

The cell cycle is a highly controlled process that is regulated by the expression of cyclins, cyclin-dependent kinases (CDKs), inhibitors of CDKs and tumor suppressors (p53, Rb). Many tumor cells overexpress CDKs increasing their proliferative capacity. CDK-cyclin complexes tightly monitor the four sequential phases of cell cycle, namely G1, S, G2, and M phase, and dysregulation in any phase results in uncontrolled cell growth (Asghar et al., 2015). Overexpression of CDK4 found to be linked to the development of paclitaxel resistance in ovarian cancer cells. Recently, Gao et al. reported that inhibition of CDK4 by palbociclib resensitized both Rb-positive and Rb-negative MDR ovarian cancer cells with paclitaxel by increasing apoptosis (Gao et al., 2017). In this study, genes involved in cell cycle regulation appeared as a determinant of BetA's activity in the NCI cell lines. This coincides with previous studies showing that BetA arrested the cell cycle at the G1 phase, induced apoptosis through the mitochondrial pathway, and inhibited angiogenesis in breast cancer cells (Damle et al., 2013; Hsu et al., 2015). The mechanisms by which BetA induced cell cycle arrest included increased expression of p53 and p21 (Foo et al., 2015), mitochondrial perturbations (Fulda and Kroemer, 2009), and downregulation of anti-apoptotic Bcl-2 family proteins (Bcl-2, Bcl-XL) (Foo et al., 2015).

Signal transducers are frequently mutated in tumor cells, playing a significant role in tumorigenesis and drug resistance (Quintás-Cardama and Verstovsek, 2013). BetA downregulated the activation of STAT3 through the upregulation of Src homology 2 domain-containing phosphatase 1 (SHP-1), and affected the STAT3/HIF-1/VEGF signal pathway (Shin et al., 2011). Therefore, it is not surprising that the STAT3 upstream signal activator, *ERBB3*, and protein phosphatase 2A activator, *PPP2R4*, appeared as molecular determinants for BetA's activity in our COMPARE analysis.

Modulation of chromatin is essential for cellular proliferation and deregulation in the degree of compaction of chromatin plays a pivotal role in the control of gene expression, replication, and repair and of chromosome segregation (Croce, 2008). The basic skeleton blocks of chromatin are nucleosomes that are composed of 146 base pairs of DNA wrapped around an octamer containing two each of four core DNA packaging proteins, i.e., the histones H2A, H2B, H3, and H4. The nucleosomes are further folded with the aid of linker histone H1 and non-histone proteins into an ordered, compact nucleoprotein complex (Thomas and Kornberg, 1975; Saha et al., 2006). Chromatin is a critical regulator of transcription and tumor suppressor genes. Therefore, deregulation of chromatin leads to genes activation and/or inappropriate genes silencing (Nair and Kumar, 2012). In our analysis, genes involved in chromatin remodeling and tumor suppression were identified as sensitivity determinants to BetA.

Furthermore, cell adhesion genes appeared as sensitivity determinants for BetA. Loss of cell-cell adhesive interaction represents an initiation step for invasion and metastasis of malignancies. It has been reported that BetA inhibits aminopeptidase N, which is tightly associated with extracellular matrix components and involved in tumor cell invasion and metastatic activity during tumor development (Melzig and Bormann, 1998; Karna et al., 2010).

In conclusion, BetA exhibited remarkable cytotoxic activity against MDR cell lines. As shown in this study, the clinically established MDR conferring proteins (P-gp, BCRP, ABCB5 and ΔEGFR) did not hamper BetA activity in tumor cells. Furthermore, microarray-based expression profiling of 60 NCI cell lines revealed that BetA exerts cytotoxic activity toward cancer cells by multiple mechanisms rather than by a single one. Summing up all findings in this study, we can postulate that BetA is a promising candidate with multiple modes of action to treat refractory tumors. Further investigations are needed to assure its efficacy and safety *in vivo*.

## Author contributions

MS: carried out the *in vitro* experiments and wrote the manuscript; NM: performed COMPARE and cluster analysis; YS: provided ABCB5 transfected cell lines; HA-A: designed the bioassays and edited the manuscript; TE: supervised the work, provided the facilities for the study and edited the manuscript. All authors read the manuscript and approved the final version.

### Conflict of interest statement

HA-A was employed by Steigerwald, Darmstadt, Germany. The other authors declare that the research was conducted in the absence of any commercial or financial relationships that could be construed as a potential conflict of interest.
